# Assessing Viscosity in Sustainable Deep Eutectic Solvents and Cosolvent Mixtures: An Artificial Neural Network-Based Molecular Approach

**DOI:** 10.1021/acssuschemeng.3c07219

**Published:** 2024-05-13

**Authors:** Luan Vittor Tavares Duarte de Alencar, Sabrina Belén Rodríguez-Reartes, Frederico Wanderley Tavares, Fèlix Llovell

**Affiliations:** † Department of Chemical Engineering, ETSEQ, Universitat Rovira i Virgili, Avinguda Països Catalans 26, 43007 Tarragona, Spain; ‡ Programa de Engenharia Química (PEQ/COPPE), Universidade Federal do Rio de Janeiro (UFRJ), Athos da Silveira Ramos Avenue, 149 - Block G -Ilha do Fundão, Rio de Janeiro, RJ 21949-900, Brazil; § Departamento de Ingeniería Química, Universidad Nacional del Sur (UNS), Avda. Alem 1253, Bahía Blanca 8000, Argentina; ∥ Planta Piloto de Ingeniería Química − PLAPIQUI (UNS-CONICET), Camino “La Carrindanga” Km 7, Bahía Blanca 8000, Argentina; ⊥ Engenharia de Processos Químicos e Bioquímicos, Escola de Química (EPQB), Universidade Federal do Rio de Janeiro (UFRJ), Athos da Silveira Ramos Avenue, 149 - Block E - Ilha do Fundão, Rio de Janeiro, RJ 21949-900, Brazil

**Keywords:** deep eutectic solvents, viscosity, machine learning, artificial neural network, COSMO-SAC

## Abstract

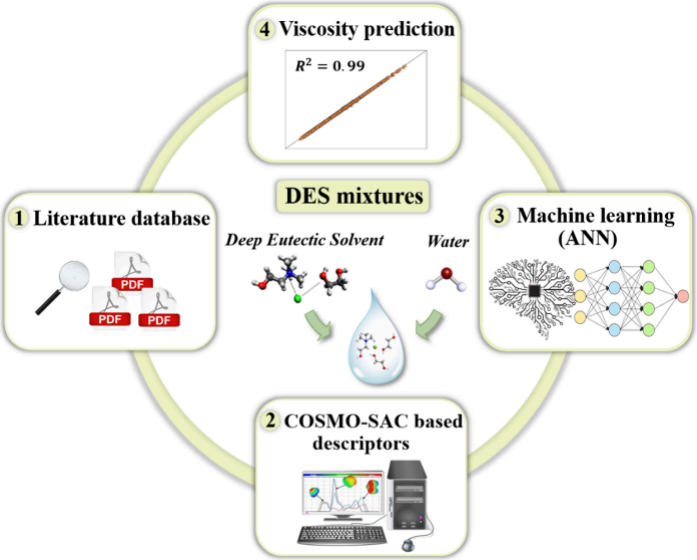

Deep eutectic solvents (DESs) are gaining recognition as environmentally friendly solvent alternatives for diverse chemical processes. Yet, designing DESs tailored to specific applications is a resource-intensive task, which requires an accurate estimation of their physicochemical properties. Among them, viscosity is crucial, as it often dictates a DES’s suitability as a solvent. In this study, an artificial neural network (ANN) is introduced to accurately describe the viscosity of DESs and their mixtures with cosolvents. The ANN utilizes molecular parameters derived from σ-profiles, computed using the conductor-like screening model for the real solvent segment activity coefficient (COSMO-SAC). The data set comprises 1891 experimental viscosity measurements for 48 DESs based on choline chloride, encompassing 279 different compositions, along with 1618 data points of DES mixtures with cosolvents as water, methanol, isopropanol, and dimethyl sulfoxide, covering a wide range of viscosity measurements from 0.3862 to 4722 mPa s. The optimal ANN structure for describing the logarithmic viscosity of DESs is configured as 9-19-16-1, achieving an overall average absolute relative deviation of 1.6031%. More importantly, the ANN shows a remarkable extrapolation capacity, as it is capable of predicting the viscosity of systems including solvents (ethanol) and hydrogen bond donors (2,3-butanediol) not considered in the training. The ANN model also demonstrates an extensive applicability domain, covering 94.17% of the entire database. These achievements represent a significant step forward in developing robust, open source, and highly accurate models for DESs using molecular descriptors.

## Abstract 2

By using machine learning to predict the viscosity of eco-friendly deep eutectic solvents, this research advances DES implementation in sustainable processes.

## 1 Introduction

Organic solvents have played a crucial role in several industrial applications such as pharmaceuticals, paints, cosmetics, biochemistry, and food. Nevertheless, most classical organic solvents are harmful and toxic, generating worldwide concern about the damage they are causing to the environment and human beings. Therefore, the search for eco-friendly and viable alternative solvents to replace conventional organic compounds in chemical applications has gained attention in recent years.^1,2^ In this context, deep eutectic solvents (DESs) have risen as promising novel “green” solvents because they can present attractive environmentally friendly properties, such as high biodegradability, low toxicity, low volatility, nonflammability, and chemical stability.^3,4^

First reported in 2003 by Abbott et al.,^5^ DESs are eutectic mixtures composed of two or more hydrogen bonding components, which have significant depressions in melting points due to hydrogen bond interactions between the hydrogen bond donor (HBD) and the hydrogen bond acceptor (HBA).^6,7^ Various components can act as HBD or HBA in the formation of DESs. In general, the choline chloride ([Ch]Cl) quaternary ammonium salt is the most widely used HBA to prepare DESs, due to its low cost, nontoxicity, biodegradability, biocompatibility, and economic synthesis.^6,8,9^ This salt is considered an essential nutrient that can be extracted from biomass and is regarded as a part of B-complex vitamins.^10^ Concerning HBD, the DESs are usually based on polyalcohols, polyacids, or polyamines.^6,11,12^

The physical and chemical properties of DESs depend on their individual constituents and HBD:HBA ratio, since the vast hydrogen bond network directly affects the characteristics of these mixtures.^3,13,14^ Thus, by adjusting the molar ratio and types of HBAs and HBDs, the physicochemical properties of DESs can also be tuned to give DESs a wide range of applicability, including material synthesis,^15−17^ separation processes,^18−21^ nanotechnology,^22,23^ biotechnology,^24,25^ and pharmaceutical processing.^26^

Nonetheless, when it comes to the industrial-scale implementation of DESs, a deep understanding of their thermophysical properties is required.^27,28^ In this regard, viscosity is a critical fundamental property that generally governs the use of DESs as a solvent in chemical processes, since it influences the fluid flow, mass transfer, and heat transfer, affecting their suitability for particular applications.^8,29,30^ However, the majority of DESs exhibits elevated viscosity at room temperature, primarily due to the hydrogen bond network existing among their constituents.^31^ This characteristic can prevent its industrial application in different fields.^32,33^ A practical approach to overcome this obstacle is adding water or other cosolvents in controlled amounts to DESs, once this has proven to significantly reduce the viscosity of the resulting eutectic mixture.^31−35^

With a vast array of potential DESs combinations, including mixtures with cosolvents under various industrial conditions (e.g., molar ratio, temperature, and pressure^36−38^), relying solely on experimental measurements for each DES’s viscosity data becomes time-consuming. Hence, developing computational models for predicting DES viscosity is paramount to streamlining their industrial implementation deployment.^28,36^ However, the viscosity behavior in mixtures of cosolvents and DESs becomes notably complex due the strong sensitivity of DESs viscosity to even minor additions of a second solvent in the mixture, where this property may change several orders of magnitude. For instance, Yadav and Pandey^30^ showed that a mere 2.25% weight of water added to [Ch]Cl: Urea (1:2) DES induced a significant 33.27% viscosity reduction (from 1003.94 to 669.90 mPa s at 293.15 K and 0.1 MPa). This sensitivity makes describing the viscosity in DESs and cosolvent mixtures more difficult than other properties.

In recent years, the application of different computational tools to predict the viscosity of DESs has been explored in several papers.^39−51^ To cite some interesting studies, Mjalli and Naser^39^ have evaluated the applicability of the Eyring-Wilson and the Vogel–Fulcher–Tamman equations to describe the viscosities of nine [Ch]Cl-based DESs. Their models accounted for both, the temperature and the salt mole fraction, resulting in *R*^2^ values greater than 0.992 for both equations. Lloret et al.^42^ applied the free volume theory (FVT) coupled into the soft-SAFT equation of state for describing the viscosity of ten tetraalkylammonium chloride-based DESs. DESs were modeled using two approaches: one treated them as a pseudopure compound, and the other described them as a mixture of two constituents. Both modeling approaches generated accurate viscosity descriptions, but the second method offered greater realism and physical consistency when modeling the behavior of the DESs. This work has been recently extended to 7 choline chloride-based DESs using the FVT combined with the spider-web methodology to enhance the parametrization of all the evaluated compounds.^52^ Roosta et al.^51^ combined a group contribution method with machine learning (ML) techniques to predict the viscosities of 305 DESs using 2533 data points. Particularly, they employed the multilayer perceptron artificial neural network and least squares support vector machine. Both models exhibited average absolute relative deviations below 10% and *R*^2^ values greater than 0.98.

Certainly, ML techniques have facilitated the creation of models capable of processing complex information. In this sense, ANNs have emerged as a potent tool for modeling intricate procedures. ANNs use experimental data during the learning process to define the outcomes of a system by recognizing patterns and connections in a provided database.^53^ Several contributions in the literature have demonstrated the elevated accuracy achieved by molecular-based ANN models in predicting the physicochemical properties of DESs.^45,54−57^ Nonetheless, achieving this level of accuracy depends not only on optimizing the ANN effectively but also on selecting the appropriate input descriptors. These descriptors should be capable of capturing the molecule’s essential characteristics and leading to the accurate determination of a specific physicochemical property value. In this context, the conductor-like screening model for real solvents (COSMO-RS) and their molecular charge density distributions (σ-profile) have previously been used as input parameters in ML models to obtain highly accurate predictions of different properties of DESs, such as density,^44^ thermal conductivity,^58^ pH,^59^ surface tension,^54^ CO_2_ solubility,^60^ electrical conductivity^57,61^ and viscosity.^45^ For instance, Benguerba et al.^45^ developed an ANN model using COSMO-RS-based σ-profiles as molecular parameter inputs to predict the viscosity of five amine-based DESs. They employed a data set containing 108 experimental data, achieving *R*^2^ values of 0.9975 and 0.9863 for the training and validation steps, respectively.

However, the use of ML techniques to quantify viscosities across wide temperature and compositional ranges for mixtures of DESs with cosolvents has received limited attention. Considering the critical role of water and other cosolvents, like alcohols, on DESs viscosity, the objective of this work is to construct an ANN model to anticipate this influence precisely. This model is designed to describe the viscosity of the prevalent sustainable [Ch]Cl-based DESs, whether in their pure form or when combined with water or other cosolvents, by employing the temperature and the compound σ-profiles as molecular descriptors, acquired through the conductor-like screening model for real solvent segment activity coefficient (COSMO-SAC). The reliability of the developed model is validated using several statistical parameters, and its predictive capability is verified by addressing new HBDs and cosolvents not included in the training, as well as calculating the applicability domain evaluation. Additionally, the influence of the molecular descriptors as input parameters on the viscosity of DESs is reported and rationally discussed.

## 2 Methodology

### 2.1 Experimental Data Set

In this study, a DESs viscosity (mPa s) database containing 1891 experimental data points was used to develop a feed-forward ANN model. The collected experimental data includes 48 different DESs mixtures based on [Ch]Cl with 18 different HBDs: phenol (PH), glycerol (GL), ethylene glycol (EG), triethylene glycol (TEG), propionic acid (PA), oxalic acid (OA), levulinic acid (LevA), glutaric acid (GA), malonic acid (MA), lactic acid (LA), p-cresol, 1,4-butanediol (1,4-BT), monoethanolamine (MEA), diethanolamine (DEA), methyldiethanolamine (MDEA), d-glucose (D-GLU), D-fructose (D-FT), and urea (UR). Additionally, the database also contains mixtures of DESs with four cosolvents: water, methanol (MeOH), isopropanol (IPA), and dimethyl sulfoxide (DMSO). The exact distribution includes 273 data points of pure DESs, and 1618 DESs + cosolvent data points, including water (894), methanol (360), isopropanol (208), and dimethyl sulfoxide (156).

Table 1 presents a comprehensive list of the DESs and mixtures considered in this study for the ANN design, along with the composition and temperature ranges for the data sets and the corresponding references.

**Table 1:** Summary of Studied DESs and DES + Cosolvents Including Their Experimental Temperature and Viscosity Ranges at Atmospheric Pressure, Number of Data Points, and Corresponding References

**DES**	**system**	**range of *T*** (K)	**range of η** (mPa s)	**no. of data points**	**ref**
DES1	[Ch]Cl + PH (1:2)	293.2–333.15	19.5–120.77	9	(62, 63)
DES2	[Ch]Cl + PH (1:3)	293.2–333.15	13.77–57.84	8	(62, 64)
DES2.1	[Ch]Cl + PH (1:3) + H_2_O	293.2–333.15	1.09–56.09	45	(64)
DES3	[Ch]Cl + PH (1:4)	293.2–318.2	14–40.23	6	(62)
DES3.1	[Ch]Cl + PH (1:4) + H_2_O	293.2–333.15	1.1–43.9	45	(64)
DES4	[Ch]Cl + PH (1:5)	293.2–318.2	11.26–31.96	6	(62)
DES5	[Ch]Cl + PH (1:6)	293.2–318.2	9.46–27.03	6	(62)
DES6	[Ch]Cl + GL (1:2)	283.15–363.15	19.59–1003.94	13	(8, 65)
DES6.1	[Ch]Cl + GL (1:2) + H_2_O	283.15–363.15	0.39–669.90	204	(8, 65)
DES6.2	[Ch]Cl + GL (1:2) + MeOH	288.15–323.15	0.43–425.75	144	(65)
DES6.3	[Ch]Cl + GL (1:2) + DMSO	288.15–323.15	1.418–497.3	104	(66)
DES6.4	[Ch]Cl + GL (1:2) + IPA	288.15–323.15	1.13–418.5	104	(66)
DES7	[Ch]Cl + GL (1:3) + H_2_O	298.15–343.15	10.98–62.05	10	(67)
DES8	[Ch]Cl + GL (1:4)	293.15–323.15	87.96–578.2	7	(68)
DES9	[Ch]Cl + EG (1:2)	283.15–323.15	17.41–87.45	9	(65)
DES9.1	[Ch]Cl + EG (1:2) + H_2_O	288.15–323.15	0.6–54.67	144	(65)
DES9.2	[Ch]Cl + EG (1:2) + MeOH	283.15–323.15	0.43–60.35	153	(65, 69)
DES9.3	[Ch]Cl + EG (1:2) + DMSO	308.15–323.15	1.40–24.88	52	(66)
DES9.4	[Ch]Cl + EG (1:2) + IPA	288.15–323.15	1.16–55.70	104	(66)
DES10	[Ch]Cl + EG (1:3)	293.15–348.15	6.79–30.17	12	(67)
DES10.1	[Ch]Cl + EG (1:3) + H_2_O	293.15–333.15	10.47–37.35	9	(70)
DES11	[Ch]Cl + EG (1:4) + H_2_O	293.15–333.15	9.01–31.8	9	(70)
DES12	[Ch]Cl + EG (1:5) + H_2_O	293.15–333.15	7.5–28.49	9	(70)
DES13	[Ch]Cl + EG (1:6) + H_2_O	293.15–333.15	6.91–25.56	9	(70)
DES14	[Ch]Cl + MA (1:0.5)	303.15–353.15	46.2–1460.3	6	(39)
DES15	[Ch]Cl + MA (1:1)	303.15–353.15	15.2–417	6	(39)
DES15.1	[Ch]Cl + MA (1:1) + H_2_O	293.15–348.15	10.14–2016	24	(29)
DES16	[Ch]Cl + MA (1:2)	303.15–353.15	30–800	6	(39)
DES17	[Ch]Cl + TEG (1:3)	298.15–358.15	9–68	7	(39)
DES18	[Ch]Cl + TEG (1:4)	298.15–358.15	8.1–61.9	7	(39)
DES19	[Ch]Cl + TEG (1:5)	298.15–358.15	7.5–53	7	(39)
DES20	[Ch]Cl + TEG (1:6)	298.15–358.15	6.5–44.9	7	(39)
DES21	[Ch]Cl + UR (1:1.5)	303.15–353.15	36.5–663	6	(39)
DES22	[Ch]Cl + UR (1:2)	293.15–363.15	19.95–1371.97	8	(30)
DES22.1	[Ch]Cl + UR (1:2) + H_2_O	293.15–363.15	0.57–436.11	72	(30)
DES23	[Ch]Cl + UR (1:2.5)	303.15–353.15	24.8–473	6	(39)
DES24	[Ch]Cl + OA (1:1)	308.15–348.15	208.3–3332	9	(29)
DES24.1	[Ch]Cl + OA (1:1) + H_2_O	293.15–348.15	10–74.18	12	(29)
DES25	[Ch]Cl + LevA (1:2)	293.15–348.15	22.23–320.6	12	(29)
DES25.1	[Ch]Cl + LevA (1:2) + H_2_O	293.15–348.15	7.21–53.39	12	(29)
DES26	[Ch]Cl + GA (1:1)	293.15–353.15	105.8–2968	13	(29)
DES26.1	[Ch]Cl + GA (1:1) + H_2_O	293.15–353.15	9.32–78.24	13	(29)
DES27	[Ch]Cl + LA (1:1)	303.15–333.15	202.46–1245.36	7	(71)
DES27.1	[Ch]Cl + LA (1:1) + H_2_O	288.15–353.15	5.53–1554.57	33	(72)
DES27.2	[Ch]Cl + LA (1:1) + MeOH	303.15–333.15	0.7–220.96	63	(71)
DES28	[Ch]Cl + LA (1:1.5) + H_2_O	288.15–353.15	4.60–4005.06	26	(72)
DES29	[Ch]Cl + LA (1:2) + H_2_O	288.15–353.15	5.43–3823.11	25	(72)
DES30	[Ch]Cl + LA (1:2.5) + H_2_O	288.15–343.15	7.08–4722.11	23	(72)
DES31	[Ch]Cl + p-cresol (1:2)	293.15–333.15	19.6–133.68	9	(63)
DES32	[Ch]Cl + 1,4-BT (1:3)	303.15–343.15	15.7–49.3	9	(73)
DES33	[Ch]Cl + 1,4-BT (1:4)	303.15–343.15	14–54.75	9	(73)
DES34	[Ch]Cl + 1,4-BT (1:5)	293.15–333.15	19.64–93.6	9	(70)
DES35	[Ch]Cl + 1,4-BT (1:6)	293.15–333.15	18.98–91.44	9	(70)
DES36	[Ch]Cl + MEA (1:5)	293.15–323.15	14.98–18.98	4	(32)
DES36.1	[Ch]Cl + MEA (1:5) + H_2_O	293.15–323.15	1.21–58.42	36	(32)
DES37	[Ch]Cl + MEA (1:6)	293.15–323.15	13.62–54.07	4	(32)
DES37.1	[Ch]Cl + MEA (1:6) + H_2_O	293.15–323.15	1.12–50.14	36	(32)
DES38	[Ch]Cl + MEA (1:8)	293.15–323.15	11.42–44.9	4	(32)
DES38.1	[Ch]Cl + MEA (1:8) + H_2_O	293.15–323.15	1.12–42.61	36	(32)
DES39	[Ch]Cl + MEA (1:10)	293.15–323.15	10.29–39.49	4	(32)
DES39.1	[Ch]Cl + MEA (1:10) + H_2_O	293.15–323.15	1.18–37.94	36	(32)
DES40	[Ch]Cl + DEA (1:6)	293.15–333.15	49.91–567	3	(55)
DES41	[Ch]Cl + DEA (1:8)	293.15–333.15	53.36–565.3	3	(55)
DES42	[Ch]Cl + DEA (1:10)	293.15–333.15	54.56–611.4	3	(55)
DES43	[Ch]Cl + MDEA (1:6)	293.15–333.15	22.01–139.8	3	(55)
DES44	[Ch]Cl + MDEA (1:8)	293.15–333.15	20.99–126.3	3	(55)
DES45	[Ch]Cl + MDEA (1:10)	293.15–333.15	9.26–54.54	3	(55)
DES46	[Ch]Cl + D-GLU (1:1) + H_2_O	293.15–353.15	52.01–2509.57	13	(74)
DES47	[Ch]Cl + D-FT (1:1) + H_2_O	293.15–353.15	28.99–995.34	13	(74)
DES48	[Ch]Cl + PA (1:2)	288.15–338.15	13.53–91.74	11	(75)

The data set covers a wide range of viscosity measurements (0.3862–4722 mPa s) and temperatures (283.15–363.15 K), with data containing 279 systems of different compositions at atmospheric pressure for binary and ternary mixtures. The complete data set is provided in full detail in Table S1 in the Supporting Information.

### 2.2 Molecular Inputs

**Figure 1:**
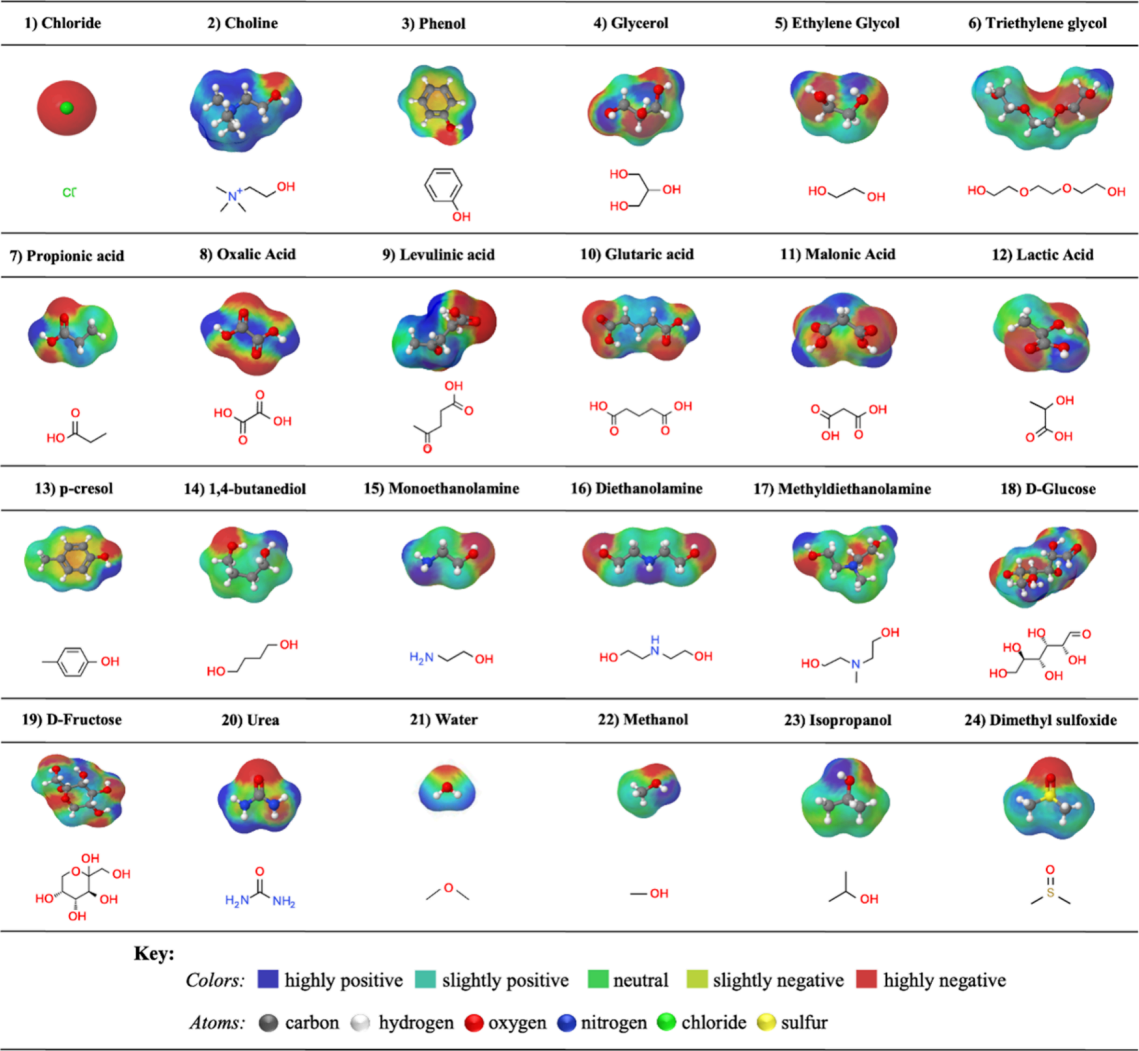
3D geometrically optimized COSMO-SAC surfaces and 2D molecular structures of the compounds used to form the DESs and cosolvents investigated in this work.

Here, COSMO-SAC is employed to obtain the molecular descriptors, particularly σ-profiles, representative of the investigated DESs, which are used as inputs for the ANN model. The COSMO-SAC model is a methodology based on quantum mechanics calculations that create a virtual conductor around each molecule, allowing the acquisition of the distribution of the density charge induced on the surface of the molecule.^76^ The probability of a specific charge density on a surface segment can be represented by a 2D histogram known as the σ-profile. The procedure to obtain σ-profiles in this work is done following the methodology available in the literature.^77^ All σ-profiles were taken from the open-source LVPP-sigma profile database,^78^ freely available at https://github.com/lvpp/sigma. The 2D molecular structure and geometrically optimized 3D COSMO-SAC surfaces of the 24 compounds investigated in this study are shown in Figure 1. The molecular polarity is visually represented through a spectrum of colors ranging from blue to red. Shades of blue indicate a higher positive charge (associated with hydrogen-donating areas). In contrast, deeper shades of red denote a greater negative charge (corresponding to hydrogen-accepting areas).

The σ-profile of a molecule provides valuable information about its structure, such as its polarity and the concentration of specific atoms within it. Consequently, the area under the σ-profile curves can be utilized to quantitatively represent the molecular surface, denoted as *S*_σ-profiles_.^79^ Therefore, using these molecular descriptors as ANN inputs allows us to establish a connection between the molecular structure and a specific property of the DES, such as the viscosity in this work.

In order to find a balance between precision and mathematical complexity of the designed neural network, this study has discretized the σ-profiles of the compounds investigated into eight distinct regions, as similarly done by Lemaoui et al.^54,59^ and Alkhatib et al.^80^ The areas under the σ-profile curves within each of these delineated regions were computed through the trapezoidal rule, and their resulting numerical area values were then employed as the *S*_σ-profiles_ of the 24 compounds investigated. Subsequently, the *S*_σ-profiles_ of the modeled DESs and their mixtures with cosolvents were computed through the conventional approach employed in the literature,^54,57,59,61^ which involves calculating the molar-weighted average of its constituents:
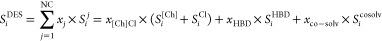
1where NC is the total number of components in the DES mixture, *x*_*j*_ is the mole fraction of component *j* in the mixture, and *S*_*i*_^*j*^ is the *S*_σ-profiles_ of component *j* in region *i,* from 1 to 8 (e/Å). *x*_[Ch]Cl_, *x*_HBD_ and *x*_cosolv_represent the molar fractions of [Ch]Cl, the HBD, and the cosolvent present within the DES, respectively.

### 2.3 Development of the ANN Model

In this work, an artificial neural network (ANN) algorithm was selected to relate the molecular descriptors to the viscosity of the DESs. Details on the conception of ANNs are fully described in the literature.^81,82^ The ANN design was conducted using the neural network toolbox of MATLAB R2023a software, employing the Bayesian regularization algorithm (*trainbr*) as the training function for the ANN. The eight *S*_σ-profiles_ and the temperature descriptor in K (*T*) were employed as the ANN input to predict the log_10_ viscosity (η) of the DESs (in this work denoted as log (η)) and their mixtures as an output response. The reason behind computing the logarithm instead of the direct property is to homogenize the weight when computing the errors of low viscosity compared to high viscosity data values. The predictive correlation is expressed as
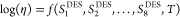
2

The typical process of constructing a neural network for a specific task involves optimization of the network structure. Therefore, to propose an effective design of the ANN model, this investigation has explored several network configurations, including single and double hidden layers with varying neuron quantities, in a similar manner as done by other authors.^83^

The hidden neurons in the neural network (*H*_*p*,*n*_) mathematical responses are expressed as follows:^55^
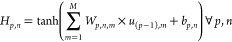
3

The subscript *p* denotes the hidden layer number (i.e., 1 or 2); thus, *H*_1,*n*_ and *H*_2,*n*_ indicate the neurons in hidden layers 1 and 2, respectively. *W* represents the weight coefficient corresponding to the connection between the input of the layer (*u*_(*p*–1),*m*_) and the hidden neuron, *b* represents the bias of the neuron, and the subscripts *m* and *n* indicate the number of the weight coefficient and the number of the neuron, respectively. Thus, each of the hidden layers communicates with its adjacent layers. To that end, each neuron, *n*, in the layer *p* receives *m* inputs (*u*_(*p*–1),*m*_) from the previous layer (*m* is the number of neurons in the previous layer), is multiplied by their corresponding weights (*W*_*n*,*p*,*m*_), and the bias (*b*_*n*,*p*_) is added. Then, the hyperbolic tangent of the obtained value is calculated to normalize the neuron output. The hyperbolic tangent (tanh) function in eq 3 is the activation function, which limits the neuron values between +1 and −1 to indicate activation and deactivation, correspondingly. Finally, from eq 4, the output response (log η) of the ANN model is expressed as follows:^55^
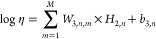
4

### 2.4 Model Evaluation

To evaluate and ensure the prediction power of the designed ANN, the database consisting of 1891 data points was split into two primary sets, a training set containing 80% of the data and a testing set containing 20%. Within the testing data set, approximately 9% was designated for internal testing during the ANN’s development, denoted as the “testing set”. In contrast, around 11% formed the “external testing set”, which remained completely untouched during the development process of the ANN. The selection of the external testing set employed the “ordered response” method,^84,85^ where the log(η) values of all DESs were sorted from lowest to highest, and then, one out of every nine data points was selected for the external testing set (see Table S2). Subsequently, the remaining data (see Table S3) was randomly divided into training and testing sets for the ANN’s development. This meticulous approach not only enhances the credibility of the model’s performance evaluation but also underscores its ability to generalize effectively to unseen data.

Furthermore, a comprehensive statistical analysis was conducted, considering classical metrics, such as the coefficient of determination (*R*^2^) to assess the linear correlation between the calculated and the experimental data, the root-mean-square error (RMSE) to measure the data dispersion around the zero deviation, and the average absolute relative deviation (AARD) to evaluate the relative absolute deviation from the experimental data.^86^ These metrics were determined using the following equations:
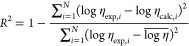
5
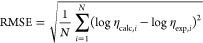
6
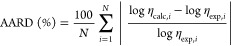
7

In these equations, log η_calc_, log η_exp_, and  represent the calculated, the experimental, and the average value of the logarithm of DES viscosities, respectively; *i* represent the specific data point considered, and *N* indicates the total number of data points.

Furthermore, to define the range of molecules in which the model prediction may be considered reliable, an applicability domain (AD) analysis was carried out. The AD of the developed ANN model was analyzed by means of the William plot,^87^ which is constructed by plotting the standardized residual (SDR_*i*_) against the leverage value (*h*_*i*_) of each data point *i*, with AD boundaries defined as horizontal boundaries (−3 < SDR < +3) and vertical boundaries (0 < *h*_*i*_ < h*), where h* denotes the critical leverage value. The points located outside the AD boundaries are treated as outliers, and their presence is attributed to variations in the chemical structure compared to the selected data points of the structural centroid used in the training set.^87^ The *h*_*i*_ and h*^88^ and the SDR_*i*_ are expressed as follows:
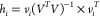
8
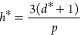
9
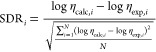
10with d* being the number of inputs within the ANN model, which is 9 in this study, *v*_*i*_ is a matrix with dimensions of 1 × d*, and *V* is a matrix with dimensions *p* × d*, where *p* indicates the number of experimental data points in training. The superscript *T* denotes the transposition of the matrices. Lastly, the coverage of the AD in a William plot can be characterized by eq 11, where *N*_inside_ represents the total number of data points within the boundaries of the AD, while *N* denotes the entire number of data points (including both the training and testing set).
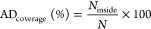
11

## 3 Results

### 3.1 Analysis of the σ-Profiles

**Figure 2:**
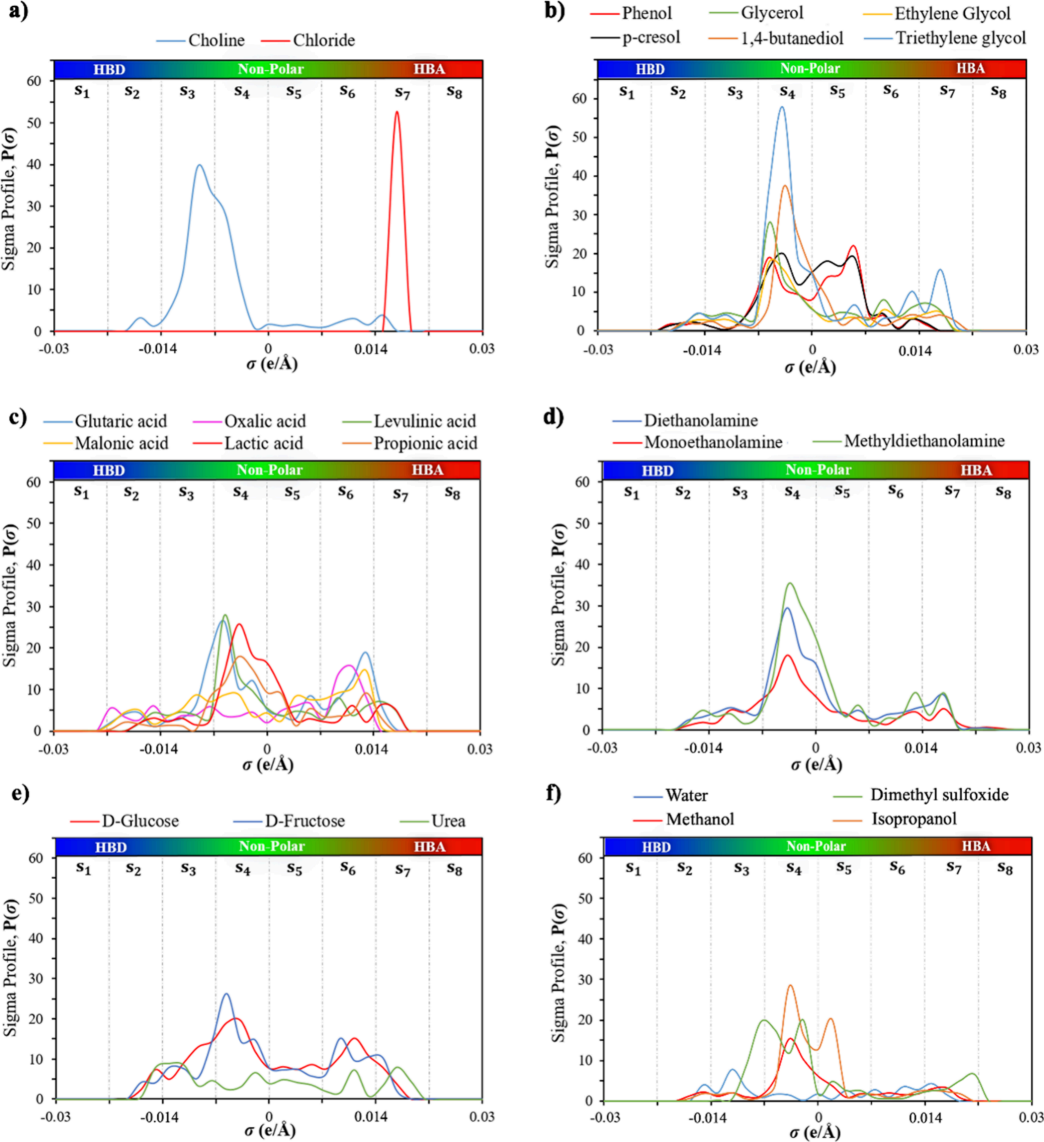
COSMO-SAC calculated σ-profiles for (a) anion and cation of the HBA [Ch]Cl salt, (b) alcohols and glycols, (c) acids, (d) amines, (e) carbohydrates and urea, and (f) cosolvents.

The discretized σ-profiles of the 24 compounds investigated were obtained through the COSMO-SAC methodology and processed according to the approach discussed in Section 2.2. The results are graphically represented in Figure 2, and the numerical values of the σ-profiles of all modeled compounds are available in Table S4 of the Supporting Information.

The sigma-profiles obtained from COSMO-SAC^77^ methodology contain 31 data points in the range of ±0.03 (e/Å). By analyzing Figure 2, it is possible to observe different regions of *S*_σ-profiles_ based on their polarized charge density: the strong hydrogen-bonding donor (HBD) region [*S*_1_ and *S*_2_], the weak HBD region [*S*_3_], the nonpolar region [*S*_4_ and *S*_5_], the weak hydrogen bond acceptor (HBA) region [*S*_6_], and the strong HBA region [*S*_7_ and *S*_8_].

The conversion of sigma-profiles into eight *S*_σ-profiles_ descriptors offers essential insights into the atomistic properties of each compound and their influence on governing intermolecular interactions by analysis of peaks in specific molecular descriptors. For instance, the peaks observed in the [*S*_4_] and [*S*_5_] zone (nonpolar region) can be attributed to the nonpolar alkyl groups present within the molecules, such as −CH_3_, −CH_2,_ and −CH. Figure 2b demonstrates that longer molecular chain lengths result in higher peak elevations in the nonpolar region (e.g., triethylene glycol > 1,4 butanediol > ethylene glycol). The peaks observed within the [*S*_1_–*S*_3_] zone mainly correspond to the positively charged H^δ+^ part of the molecules, which induces a shift toward the negative pole of the field. Conversely, within the [*S*_6_–*S*_8_] zone, the peaks are primarily associated with the electronegative regions of O^δ−^, N^δ−^, or S^δ−^ found in the O–H, N–H, and S=O groups or to the anion [Cl]^−^, which exhibits a stronger screening charge density in [*S*_7_] zone, as illustrated in Figure 2a. The calculated areas below the *S*_σ-profiles_, providing the [*S*_1_–*S*_8_] descriptors for the compounds investigated, are listed in Table S5 of the Supporting Information.

### 3.2 ANN Model: Design and Evaluation

#### 3.2.1 Optimization of the ANN Structure

**Figure 3:**
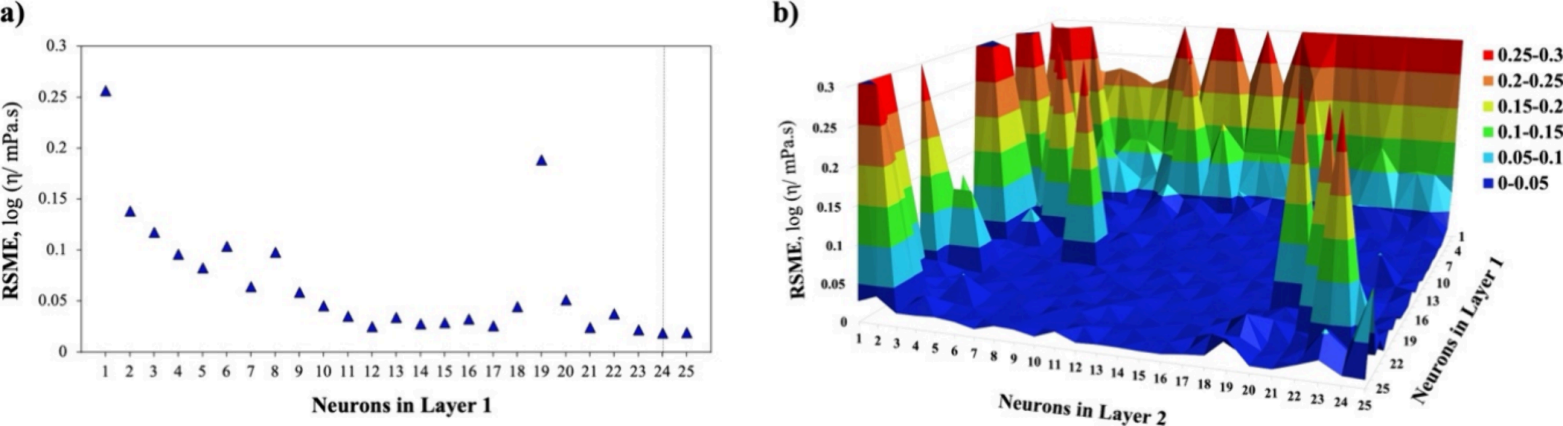
Effect of the number of neurons on the RMSE for predicting the viscosity of DES using the Bayesian regularization algorithm for training ANNs, including (a) one hidden layer and (b) two hidden layers.

The performance of an ANN model is significantly influenced by the number of neurons in the hidden layer, which exerts a considerable impact on the complexity and accuracy of the resulting models.^89^ Insufficient neurons in the hidden layer can generate an underfitted model, resulting in lower accuracy in the training and testing data. Conversely, an excessive number of neurons may lead to overfitting, wherein the model achieves a high training accuracy but poorer performance on testing data. Therefore, selecting the appropriate number of neurons in the hidden layer is pivotal for optimal ANN model performance. As a first attempt, several network structures with a single hidden layer were examined, each one employing various neurons ranging from 1 to 25. Figure 3a illustrates the influence of varying the number of neurons in the first hidden layer on the RMSE values. Notably, the figure shows that the ANN model with 24 neurons achieved the most favorable performance in predicting the viscosity logarithm of DES, achieving an RMSE value of 0.01954.

Previous literature has shown that highly nonlinear relationships tend to be more accurately modeled using ANNs with two or more hidden layers.^54,57,90^ Based on this information, an extensive analysis was performed, exploring the impact of adding a second hidden layer to the ANN. For that purpose, two layers of ANNs were designed, spanning from 1 to 25 neurons in each layer, and the performance of each network was assessed in terms of RMSE and complexity of the model.Figure 3b shows the RMSE results from examining 625 two-hidden layer configurations. Among these configurations, the ANN featuring 19 neurons in the first hidden layer and 16 neurons in the second hidden layer has been selected as the optimal compromise between accuracy and architecture complexity, as it is the simplest model that achieves one of the lowest RMSE, obtaining a value of 0.01424 in predicting the logarithm of DES viscosity in the total training and testing set. This RMSE is approximately 27% lower than the RMSE obtained by a model with a single hidden layer consisting of 24 neurons (0.01954).

**Figure 4:**
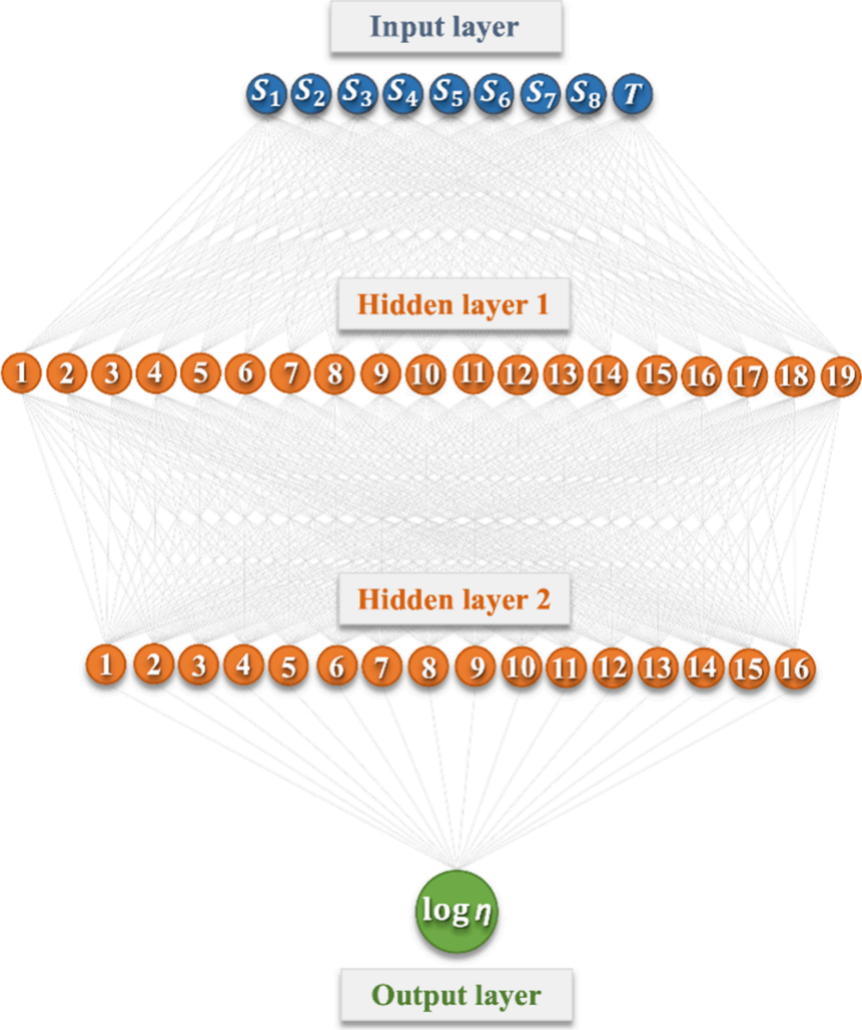
Schematic diagram of the optimal ANN model with a (9-19-16-1) configuration.

Hence, it has been determined that the most effective architecture for predicting the provided data set is 9-19-16-1. The schematic diagram of the optimal ANN is displayed in Figure 4.

**Figure 5:**
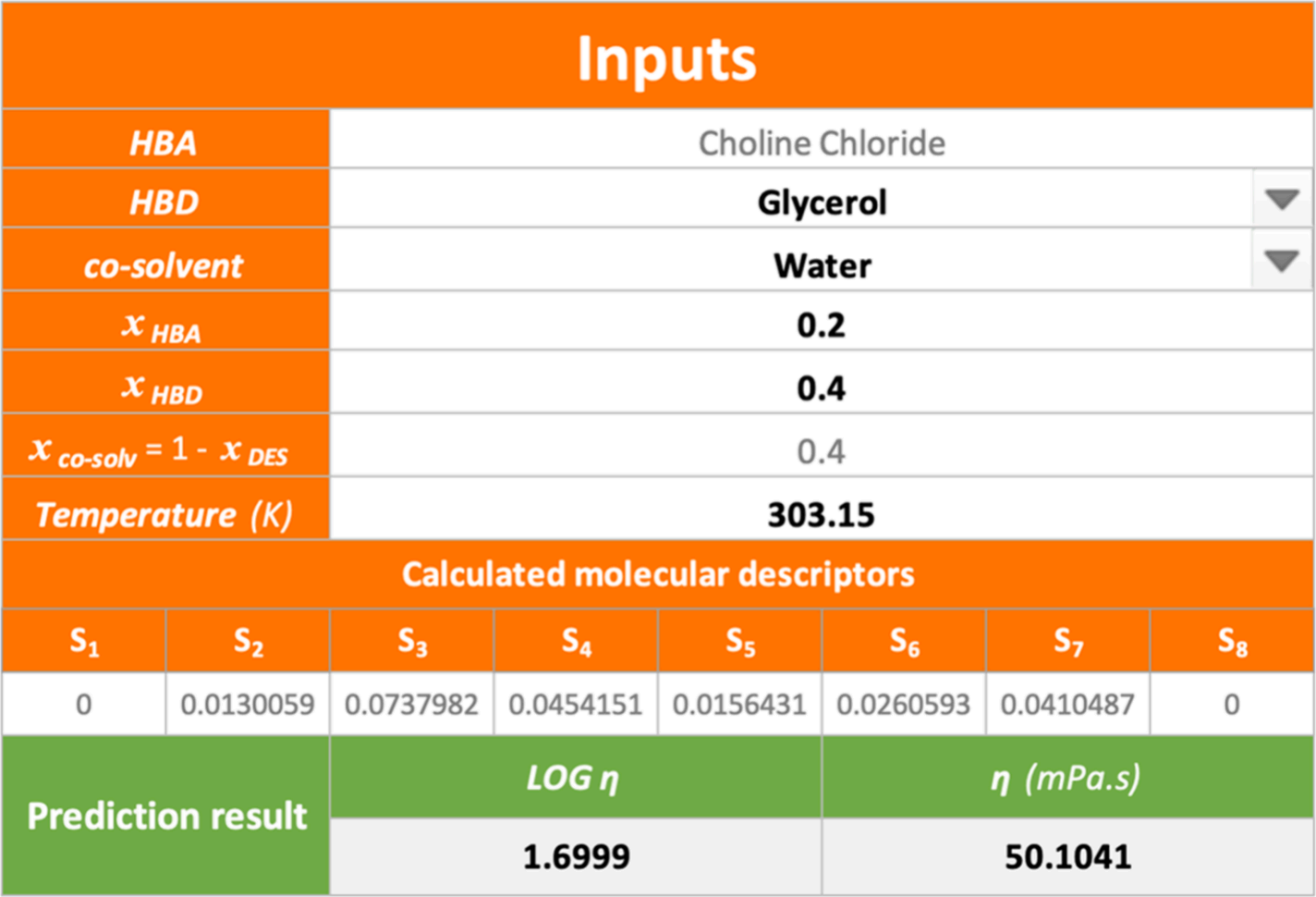
Image of the Excel spreadsheet for the prediction of viscosity of DES6.1 using Table S7.

The weights and biases of each neuron are reported in Table S6 (see the Supporting Information). Additionally, the developed ANN has been integrated into an open source and user-friendly Excel spreadsheet in Table S7 of the Supporting Information, which is showcased in Figure 5 for the prediction of viscosity of DES6.1 in Table 1. In case a new HBD or cosolvent is considered, their *S*_σ-profiles_ can be generated using the method reported in this study, and their sigma-profile can be added in Table S4. Thus, this information is automatically included in the calculator provided in Table S7 of the Supporting Information, for obtaining further viscosities predictions.

#### 3.2.2 Error Analyses

Following the determination of the optimal ANN configuration, the performance of the developed model in predicting the training and testing sets was analyzed by the assessment of various statistical parameters. A detailed summary is provided in Table S8.

The ANN model exhibits high statistical performances, achieving an *R*^2^ value of 0.99989 and an AARD value of 1.6288% for the training set, 0.99723 and 1.4729% for the testing set, and 0.99809 and 1.5225% for the external testing set. Furthermore, the RMSE values for predicting log η were also quite low, standing at 0.008271, 0.037594, and 0.035381 for the training, testing, and external testing sets, respectively, providing further evidence of the ANN model reliability.

**Figure 6:**
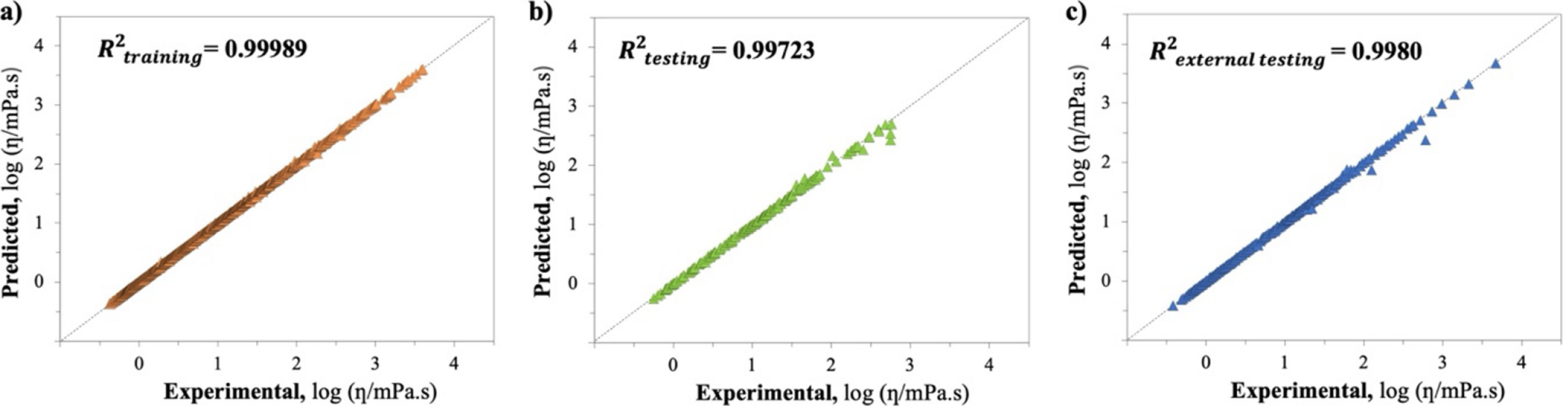
Parity graph comparing experimental and predicted DESs log η values from the ANN model, with corresponding *R*^2^ values from (a) training, (b) testing, and (c) external testing.

Figure 6 displays scatter plots of experimental and predicted DESs log η values, demonstrating the excellent model fit and absence of overfitting with most points closely aligned along the *y* = *x* diagonal.

The residual plot was also employed to analyze the model’s accuracy for further evaluation. The proposed model exhibited exceptional performance in predicting the log η of DESs, with 99.15% of residuals concentrated within the ±0.05 range (see Figure S1 in the Supporting Information). In addition, to ensure the robustness of the selected descriptors and their insensitivity to changes in the input data set, a cross-validation test based on the leave-one-system-out technique^59^ was conducted. As we aim to assess the predictivity of a thermophysical property, in an effort to obtain a statistically more meaningful value for the prediction accuracy, the cross-validation is performed for systems rather than individual data points or random sets of points, in a similar manner as done for QSAR models in a recent contribution, where this validation was done by anions.^91^ Thus, this technique is computed here by excluding one DES from the training set and determining the model’s internal fit assessed by the coefficient of determination calculated for the DES “predicted as new” by the developed model. The process is then repeated multiple times until all of the DESs shown in Table 1 are held out once from the training set, and an average of the internal fits is computed as the Q^2^ cross-validation coefficient. The mean value of *Q*^2^ was found to be 0.9044, which reflects the robustness of the model. Considering all error analyses, the developed ANN, based on COSMO-SAC σ-profiles of the compounds and the experimental data temperature, has demonstrated its ability to accurately describe DESs and DESs + cosolvent viscosities with very modest deviations.

#### 3.2.3 Predictive Capabilities

The predictive capabilities of the model are demonstrated through the application of the developed ANN to predict the viscosities of some DESs in specific conditions not included in the experimental data set. This includes scenarios featuring different combinations of HBDs and cosolvents, which were outside the scope of both the training and testing procedures. The detailed data set of these extrapolated points is available in Table S9 in the Supporting Information.

**Figure 7:**
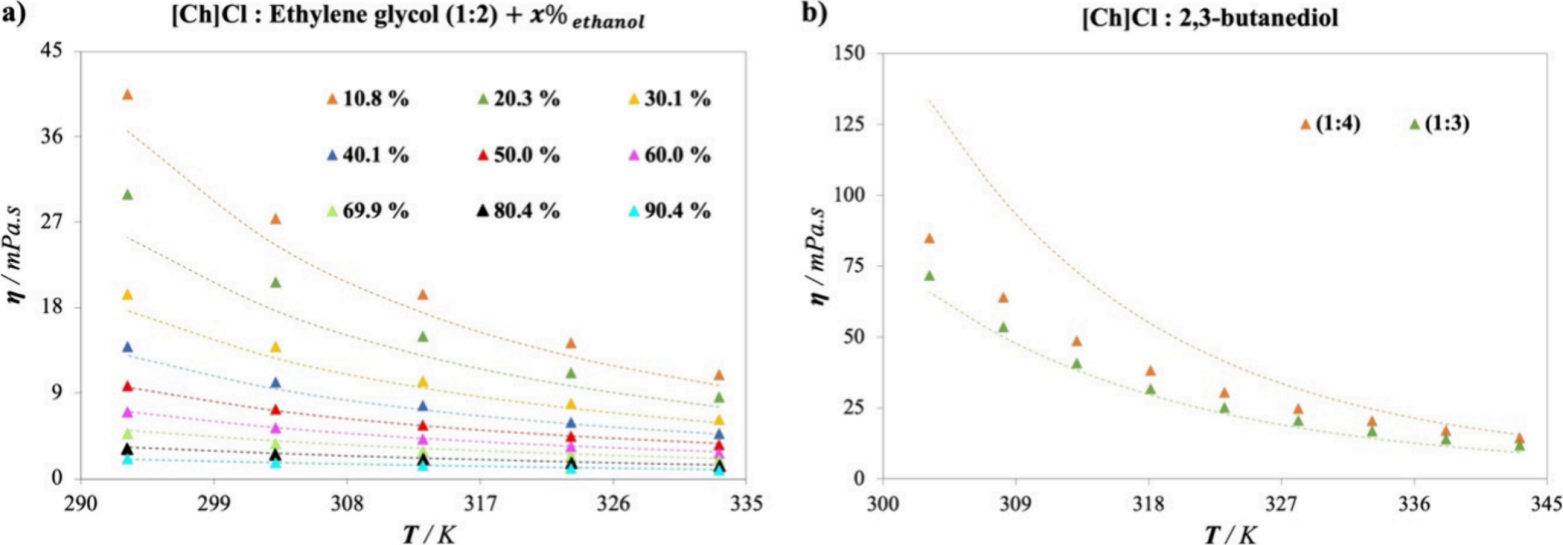
Viscosity predictions in the presence of new cosolvents or HBDs: Viscosity experimental data^29,69,73^ (symbols) and predictions calculated (dashed lines) by the proposed optimized ANN model (9-19-16-1) for (a) [Ch]Cl- EG (1:2) + ethanol, (b) [Ch]Cl- 2,3-butanediol at different proportions.

The first test encompasses the use of ethanol as a new cosolvent, through mixtures of [Ch]Cl: EG (1:2) at various ratios.^69^ The comparison between the experimental data and extrapolated predictions of DESs viscosities using the proposed ANN is depicted in Figure 7a. The high accuracy achieved by the network to predict the viscosity of [Ch]Cl:EG (1:2) in solution with ethanol is demonstrated through the low AARD of 6.19% encountered across a wide range of ethanol proportions, spanning from 10 to 90% of the molar fraction. It is important to remark that no degeneracy is observed when increasing the amount of cosolvent in the solution.

The second test explores the addition of a new HBD to the system. In this case, the viscosity of 2,3 butanediol, combined with [Ch]Cl at different ratios,^73^ is predicted in reasonable agreement with the available experimental data, as shown in Figure 7b. While the results offer an acceptable AARD of 18.91%, the major deviations are caused by the inclusion of a proportion with a bigger amount of 2,3-butanediol (1:4), increasing at lower temperatures. On the other hand, the results for [Ch]Cl:2,3-butanediol (1:3) are excellent over the whole range of temperature.

**Figure 8:**
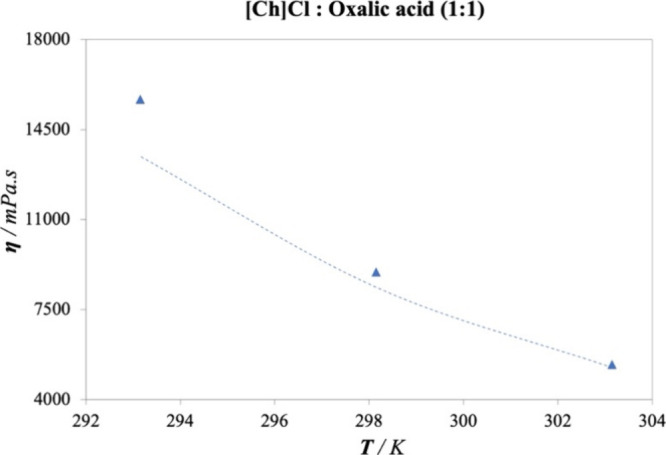
Extrapolation capability of the ANN for data outside the fitting viscosity range. Viscosity experimental data^29,69,73^ (symbols) and predictions calculated (dashed lines) by the proposed optimized ANN model (9-19-16-1) for [Ch]Cl:OA (1.1) at low temperatures.

Finally, the extrapolation capacity of the ANN is further stressed by predicting a range of extremely high viscosities, omitted from the data set due to values exceeding 5000 mPa s,^29^ for the DES [Ch]Cl: OA (1:1). The results are plotted in Figure 8. Remarkably, the ANN predicts the viscosity of [Ch]Cl: OA (1:1) with an AARD of 8.57%, covering a wide range of viscosity values from 5000 to 16,000 mPa s, highlighting the capacity to estimate values where measurements are difficult due to the inherent complexities of a highly viscous flow.

#### 3.2.4 Applicability Domain

**Figure 9:**
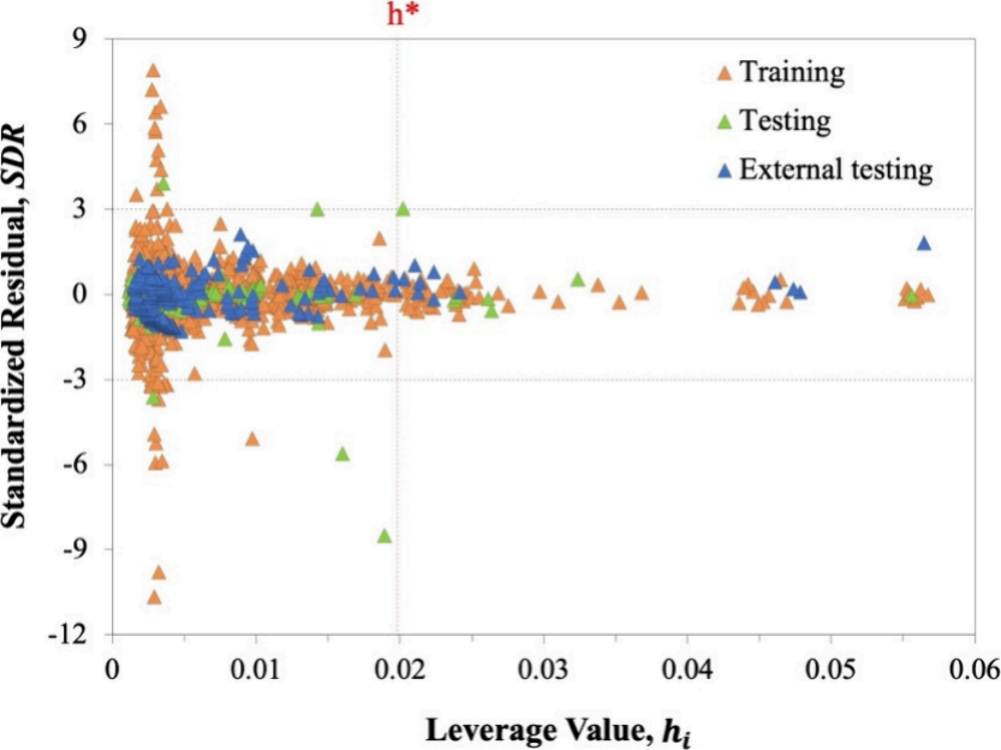
William plot for log η of the total set of DESs.

The scope and reliability of the developed ANN model can be further assessed using the applicability domain (AD) analysis. Evaluating the AD holds significant importance, especially for molecular-based ANNs, as it quantitatively defines the range of molecules and conditions where the prediction can be performed accurately.^58,59,88^ The AD of the developed ANN model was assessed using the William plot, shown in Figure 9, where the AD boundaries are defined by the vertical dashed line (0 < *h*_*i*_ < h*) and the horizontal dashed lines (−3 < SDR < +3).^88^

The analysis reveals that most of the DESs data employed in the development of the ANN model and the external testing are within the AD boundaries, with an AD_coverage_ of 94.167% across the entire data set. However, the predictions in training, testing, and external testing of some few data points at exceptional temperature values are considered structural outliers due to their leverage values surpassing h* or SDRs exceeding the standard limit of ±3. Nevertheless, these outliers constitute a minor fraction, accounting for less than 6% of the total data points. Hence, the database used in this study does not contain a substantial number of outliers, and the developed ANN model is properly accurate within its domain of applicability, indicating the robustness and reliability of the proposed ANN model due to its large AD and structural coverage.

Additionally, the new experimental data used for the study done in Section 3.2.3 are tested for their AD, yielding a 95.45% of AD_coverage_, implying that most of them fall within the model’s applicability domain. The William plot of these data is shown in Figure S4 of the Supporting Information. Particularly, the data for [ChCl]:OA (1:1) at the lowest temperatures fall outside the AD of the model. Therefore, the developed model could be considered reliable except for the aforementioned data points, for which viscosity prediction should be taken with care.

#### 3.2.5 Molecular Descriptor Importance

**Figure 10:**
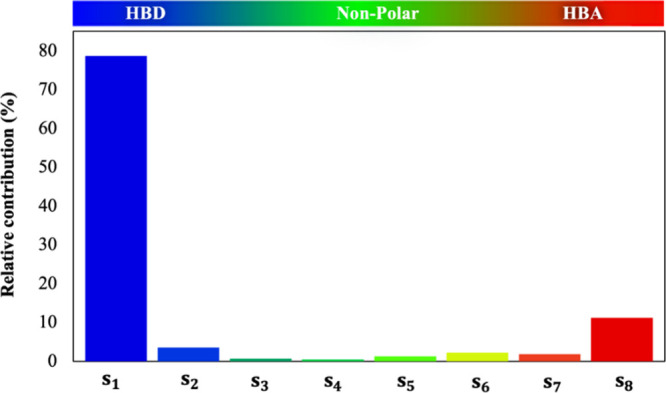
Relative contributions of the *S*_1–8_ molecular descriptor inputs to the log η of the DESs for the optimized ANN (9-19-16-1) calculated with the PaD method.

Finally, the significance of the individual input variables within the molecular-based ANN model and their influence on the viscosity of the DESs have been assessed through the performance of a relative contribution analysis. This analysis was conducted employing the partial derivatives (PaD) method, identified as the most effective approach for studying the relative contributions of input parameters to the ANN’s output.^92^ The PaD method involves computing the partial derivatives of the output over the input variables.^90^ The results of the relative contribution of the molecular descriptor inputs related to the discretization of the σ-profiles are illustrated in Figure 10.

As observed, the regions with strong hydrogen bonding areas exhibit the most significant contributions to the log η of the DESs, as the strongest HBD region [*S*_1_] presents a relative contribution of 78.71% and the strongest HBA region [*S*_8_] of 11.23%. From a chemical perspective, the viscosity of DESs primarily depends on the strength of hydrogen bonds, as the extensive network of these bonds limits the mobility of free species within the DESs, leading to a viscosity increase.^7,14^ As an example, the viscosity of [Ch]Cl-based DESs is significantly higher when using a diacid, like oxalic acid, as the HBD, compared to a monoacid, like levulinic acid, due to the formation of additional hydrogen bonds.^29^Figures S2 and S3 in the Supporting Information provide a visual representation of the direct influence of all input variables on the output. Figure S2 illustrates the relationship between derivatives of the log η with respect to each input variable and their corresponding input, and Figure S3 shows the relationship between these derivatives and the resulting log η of the DESs. Overall, the relative contributions mentioned above provide valuable insights related to the impact of hydrogen bonding on viscosity, facilitating an ad-hoc selection of promising DESs for specific applications.

## 4 Conclusions

In this work, an ANN model was developed to estimate the viscosity of DESs and their mixtures with cosolvents using COSMO-SAC-based σ-profiles and temperature as parameter inputs. The training data set included 48 DES based on [Ch]Cl with 1891 data points, encompassing 18 different HBDs and mixtures of DESs with water, methanol, isopropanol, and dimethyl sulfoxide. After evaluating 25 one-hidden and 625 two-hidden layer configurations, the ANN with the best performance in predicting the log η of DESs was found to be 9-19-16-1 architecture, achieving an RSME value of 0.01424, with high *R*^2^ values of 0.99989, 0.99723, and 0.99809 in training, internal testing, and external testing, respectively. Also, the high cross-validation coefficient (*Q*^2^ = 0.9044) suggests that the model is internally stable and robust. The extrapolative capacity of the ANN was shown by predicting the viscosity of ChCl:EG (1:2) in ethanol, a cosolvent not included in the network training, achieving remarkable agreement. In a similar manner, the ANN also demonstrated a good performance when adding a new HBD (2,3-butanediol) as well as extrapolating to viscosity values out of the trained viscosity range. Furthermore, an examination of the AD revealed that the great majority of the DESs employed in the development of the ANN model were within the AD_coverage_ of 94.17%. Finally, contribution analysis of the molecular descriptors highlighted the impact of the most charged areas of the system, establishing a direct physical connection between a strong associating/polar bond and a higher viscosity value. These results demonstrate the good performance and capacity of the ANN.

In summary, the proposed ANN model effectively captures complex input correlations, accurately describing and predicting viscosity for a wide range of [Ch]Cl-based DESs and their mixtures with different cosolvents (i.e., ethanol, isopropanol, dimethyl sulfoxide, methanol, and water), facilitating the use of these sustainable compounds into practical applications. This achievement represents a pivotal initiative in advancing the development of robust predictive models capable of estimating DESs properties, solely based on molecular descriptors. Such models hold the potential to significantly reduce the time and resources required for research, thereby accelerating the industrial-scale implementation of DESs across diverse applications.

## Supporting Information Available

Database used in this work; database used for external testing; database used for the ANN model development; numerical values of the sigma-profiles of the database compounds; calculated molecular descriptors for the database compounds; weights and biases for the optimal ANN; ANN calculator for the viscosity predictions of [Ch]Cl based DESs; statistical parameters for the performance evaluation of the developed ANN model; database used for extrapolations; illustration of the residual deviation; visual representation of the direct influence of all input variables on the output (PaD); and William plot for the extrapolation set of DESs considered (XLSX)
